# 1926. Risky businesses: Carbon dioxide monitoring to assess ventilation in grocery stores as an indicator of risk for respiratory virus transmission

**DOI:** 10.1093/ofid/ofac492.1553

**Published:** 2022-12-15

**Authors:** Maria Torres Teran, Jennifer Cadnum, Curtis Donskey

**Affiliations:** Northeast Ohio VA Medical Center, Cleveland, Ohio; Northeast Ohio VA Medical Center, Cleveland, Ohio; Cleveland VA Hospital, Cleveland, Ohio

## Abstract

**Background:**

Poorly ventilated indoor settings pose a risk for transmission of severe acute respiratory syndrome coronavirus 2 (SARS-CoV-2) and other respiratory viruses. Transmission often occurs in community, but limited information is available on the quality of ventilation in community settings.

**Methods:**

We used carbon dioxide measurements to assess adequacy of ventilation in 6 grocery stores, including large warehouse stores, medium-sized stores, and small neighborhood groceries. Carbon dioxide levels were monitored continuously with values recorded once per minute using a handheld device during multiple shopping trips to each store during both busy (defined as long lines at checkout) and non-busy (defined as no lines at checkout) shopping times. Carbon dioxide readings above 800 parts per million (ppm) were considered an indicator of suboptimal ventilation for the number of people present.

**Results:**

Carbon dioxide levels remained below 800 ppm in all 6 grocery stores during trips at non-busy shopping times, except for transient rises above 800 ppm (peak = 860 ppm) during check out in 2 stores. In 3 of the 6 stores (50%), carbon dioxide levels rose above 800 ppm for prolonged periods during busy shopping times, with considerable variability in levels in different store locations (ie, increased levels in crowded store locations). The figure shows carbon dioxide levels for 1 medium-sized grocery store during typical shopping trips during busy and non-busy shopping periods.

Increase in carbon dioxide levels in parts per million (ppm) in a grocery store during busy and non-busy shopping trips. Peak levels of carbon dioxide above 800 ppm (dotted lines) were considered an indicator of suboptimal ventilation for the number of occupants present.

**Figure:**
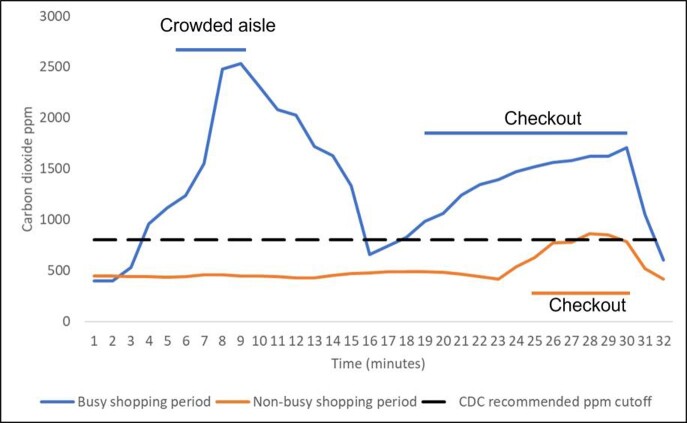

**Conclusion:**

Ventilation in grocery stores may be sufficient to minimize the risk for airborne transmission of respiratory pathogens during non-busy shopping periods, but in some stores may be sub-optimal in crowded locations during busy shopping times. There is a need for additional studies to assess the quality of ventilation in a wide range of community settings.

**Disclosures:**

**All Authors**: No reported disclosures.

